# Cuffless blood pressure estimation using only a smartphone

**DOI:** 10.1038/s41598-018-25681-5

**Published:** 2018-05-08

**Authors:** Kenta Matsumura, Peter Rolfe, Sogo Toda, Takehiro Yamakoshi

**Affiliations:** 1 Division of Bioengineering and Bioinformatics, Graduate School of Information Science and Technology, Hokkaido University, Hokkaido, Japan; 2 Computer Science Laboratory, Comprehensive Research Organization, Fukuoka Institute of Technology, Fukuoka, Japan; 3 Department of Automatic Measurement and Control, Harbin Institute of Technology, Harbin, China; 4 Oxford BioHorizons Ltd., Maidstone, United Kingdom; 5 Information and Systems Engineering, Graduate School of Engineering, Fukuoka Institute of Technology, Fukuoka, Japan; 6 Present Address: Faculty of Medicine, University of Toyama, Toyama, Japan

## Abstract

Cuffless blood pressure (BP) measurement is an all-inclusive term for a method that aims to measure BP without using a cuff. Recent cuffless technology has made it possible to estimate BP with reasonable accuracy. However, mainstream methods require an electrocardiogram and photoplethysmogram measurements, and frequent calibration procedures using a cuff sphygmomanometer. We therefore developed a far simpler cuffless method, using only heart rate (HR) and modified normalized pulse volume (mNPV) that can be measured using a smartphone, based on the knowledge that ln BP = ln cardiac output (CO) + ln total peripheral resistance (TPR), where CO and TPR are correlated with HR and mNPV, respectively. Here, we show that mean arterial pressure (MAP), systolic BP (SBP), and diastolic BP (DBP) could be estimated using the exponential transformation of linear polynomial equation, (a × ln HR) + (b × ln mNPV) + constant, using only a smartphone, with an accuracy of *R* > 0.70. This implies that our cuffless method could convert a large number of smartphones or smart watches into simplified sphygmomanometers.

## Introduction

High arterial blood pressure (BP) is a well-known risk factor for the global burden of disease and global mortality, as well as for cardiovascular diseases^1,2^. It is well established that BP varies dramatically from beat to beat, minute to minute, and year to year^3^. Thus, frequent BP checks are desirable. The direct measurement of BP requires an invasive technique, which is limited to particular clinical situations, therefore non-invasive methods are preferred in most situations. The most significant early advance in non-invasive BP measurement came in 1896 with the introduction of the ‘Riva Rocci mercury sphygmomanometer’, which was able to estimate systolic BP (SBP). With this device an inflatable rubber cuff was applied around the upper arm, and a mercury column used to measure the cuff pressure during an inflation-deflation sequence. The radial pulse was palpated to detect the pressure at which the pulse disappeared during inflation and then re-appeared during deflation, signaling SBP. The Riva Rocci cuff method, and subsequent improvements thereof, became the most widely used method for BP measurement and the BP cuff is still a very familiar sight.

There are now several non-invasive BP measurement techniques, including variants of the Riva Rocci mercury sphygmomanometry, as well as volume oscillometry ^4^, volume compensation method^5^, and tonometry^6^. When measuring BP with these sphygmomanometers, a cuff, applied around the upper arm, the wrist, or finger, is usually necessary regardless of the actual method used. However, there is increasing interest to develop techniques that do not require the use of a cuff. This could eventually signal the end of the Riva Rocci innovation as a new family of ‘cuffless’ BP technologies emerges.

‘Cuffless BP’ is now an all-inclusive term for methods that measure, or estimate, BP without using a cuff. The advantages of cuffless BP include being free from the inconvenience or pain associated with inflation of a cuff and avoiding the need for a pressurisation mechanism, which adds to the complexity and cost of the instrument. The most common method for cuffless BP is that based on the measurement of pulse wave velocity (PWV)^7–9^. This method involves taking advantage of the relationship between the BP and the PWV; the PWV is said to ‘quicken’ as the BP increases^10^. In a recent study, Ding *et al*.^11^ attained substantial accuracy by introducing new formulae. However, the PWV measurement requires a combination of an electrocardiogram (ECG) and a photoplethysmogram (PPG) e.g.^12–15^, or two PPGs e.g.^16–18^, which is inconvenient and encumbering. Furthermore, the PWV-based method usually requires an elaborate individual calibration process using a sphygmomanometer with a cuff e.g.^11,19,20^, and thus cannot be used as a replacement for a cuff sphygmomanometer^21^.

Another method that does not require an ECG or individual calibration is based on the morphology of the PPG. This measurement involves extracting the characteristics of the PPG^22–24^ and/or use of machine learning^25,26^. While this method has moderate-to-high accuracy, it still has the inconvenience of requiring the combination of a PPG and a data acquisition and transfer system, such as a bio-amplifier, Wi-Fi or Bluetooth modules, or wire connection, and a computer (or tablet) with an analogue-to-digital converter, unless it is specially designed for ambulatory measurement. Moreover, regarding machine learning, the physiological background and how to output BP data often remain unclear, so it is difficult at present to estimate the limitations and suitability of use.

Evidence from circulation physiology and psychophysiology suggests an alternative approach is possible. Mean arterial pressure (MAP) is the product of cardiac output (CO) and total peripheral resistance (TPR): MAP = CO × TPR^27^. Natural log transformation (ln) of both sides of this equation yields: ln MAP = ln CO + ln TPR. Here, CO is associated with heart rate (HR) in that both are affected by *β*-adrenergic sympathetic nerve activity^28,29^. Similarly, TPR is associated with modified normalized pulse volume (mNPV), a photoplethysmographic measure reflecting finger vascular tone^30–32^, in that both are affected by α-adrenergic sympathetic nerve activity^28–32^. Consequently, MAP should be able to be estimated using a simple linear polynomial equation: ln MAP = a × ln HR + b × ln mNPV + constant. It should be noted that both HR and mNPV are physiological variables that can be measured using a smartphone^33–35^.

Furthermore, it is also known that systolic blood pressure (SBP) is closely related to HR and CO^36,37^, whereas diastolic blood pressure (DBP) is closely related to TPR^38^. Thus, SBP should also be able to be estimated with a higher contribution of HR, and DBP with a higher weight of mNPV.

We report here a simple cuffless method for estimating MAP, SBP, and DBP using only a smartphone, as well as using a traditional finger photoplethysmograph. This is the first study that examines the accuracy of the newly proposed method.

## Results

Three data points from the iPhone 6s smartphone were treated as missing values according to the iPhysioMeter^SM^’s built-in algorithm detecting outliers^34^. One data point from the dedicated photoplethysmograph could not be analysed because of an intense artefact, and was removed. This unanalysable data point was overlapped with one of three data points treated as missing values in the smartphone data set.

### MAP, SBP, DBP, HR, and ln mNPV values during BL and MA

The mean values of each index during baseline (BL) and mental arithmetic (MA), together with other statistics, are shown in Table 1.

**Table 1:** Physiological variables during two conditions simultaneously measured by each device.

Measures	Condition		Statistics		
	BL	MA	*t*-test		Effect Size
	*M* (*SD*)	*M* (*SD*)	*t*_(12)_	*p*	*d*
Brachial sphygmomanometer					
SBP (mmHg)	117.1 (14.8)	134.6 (17.1)	7.72	<0.001	1.10
MAP (mmHg)	90.3 (10.6)	106.1 (11.7)	9.52	<0.001	1.42
DBP (mmHg)	76.9 (9.4)	91.8 (9.6)	9.27	<0.001	1.57
Smartphone					
HR (bpm)	75.0 (14.1)	92.0 (19.7)	5.71	<0.001	1.00
ln mNPV (a.u.)	−3.63 (0.35)	−4.37 (0.37)	7.87	<0.001	2.08
Laboratory photoplethysmograph					
HR (bpm)	74.9 (14.0)	92.1 (19.6)	6.02	<0.001	1.02
ln mNPV (a.u.)	−3.90 (0.49)	−4.51 (0.43)	6.50	<0.001	1.34

*Note*. SBP = systolic blood pressure, MAP = mean arterial pressure, DBP = diastolic blood pressure, HR = heart rate, mNPV = modified normalized pulse volume, BL = baseline, MA = mental arithmetic, a.u. = arbitrary unit.

### HR and ln mNPV values derived using the smartphone

**Figure 1:**
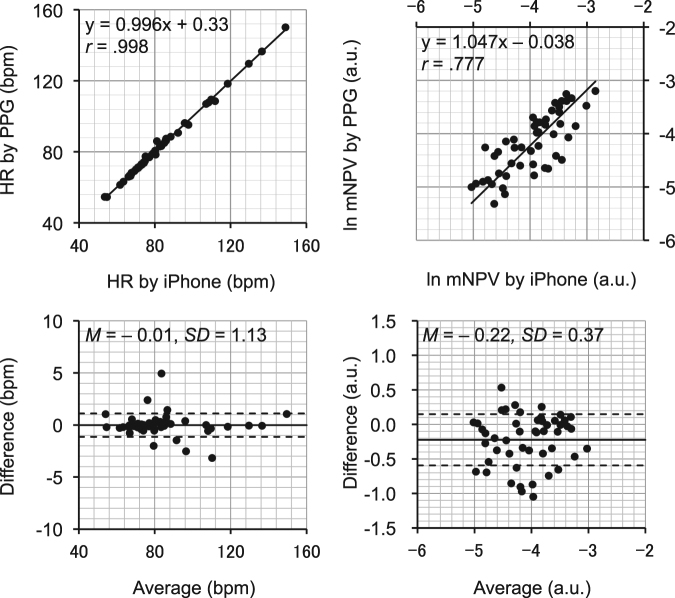
The agreement of heart rate (HR) and natural log transformation (ln) modified normalized pulse volume (mNPV) measurements derived from an iPhone 6s (iPhone) and a laboratory photoplethysmograph (PPG). (Upper) Solid line represents the geometric mean regression line and its formula, together with *r* value, is shown in each scatterplot. (Lower) Corresponding Bland–Altman plots. Solid line and dashed lines represent fixed bias (*M*) and *M* ± 1 standard deviation (SD) range, respectively. Average = (iPhone + PPG)/2, Difference = PPG – iPhone. *N* = 49.

The agreement between HR and ln mNPV measurements derived from the iPhysioMeter^SM^ run on an iPhone 6s and a laboratory photoplethysmograph with a 16-bit A/D converter are shown in Fig. 1.

### Multiple linear regression analyses

The results of multiple linear regression analyses using brachial ln MAP, ln SBP, and ln DBP as independent variables and using ln HR and ln mNPV derived using only the smartphone as dependent variables are summarized in Table 2(A). The same analyses replacing data from the smartphone with data from the dedicated photoplethysmograph are shown in Table 2(B).

**Table 2:** Multiple linear regression analyses of ln (blood pressure) = a × ln (heart rate) + b × ln (modified normalized pulse volume) + constant (c) using data from (**A**) the smartphone (*N* = 49) and (**B**) the dedicated photoplethysmograph (*N* = 51).

Dependent Variable	Coefficients								
	*R*	a			b			c	
		*β*	*std*. *β*	*p*	*β*	*std*. *β*	*p*	*β*	*p*
**A**									
ln MAP	0.688	0.305	0.472	<0.001	−0.084	−0.324	0.011	2.905	<0.001
ln SBP	0.685	0.418	0.627	<0.001	−0.029	−0.107	0.385	2.874	<0.001
ln DBP	0.685	0.220	0.327	0.010	−0.126	−0.466	<0.001	2.959	<0.001
**B**									
ln MAP	0.741	0.323	0.495	<0.001	−0.102	−0.420	<0.001	2.729	<0.001
ln SBP	0.689	0.414	0.616	<0.001	−0.043	−0.174	0.121	2.822	<0.001
ln DBP	0.774	0.255	0.373	<0.001	−0.146	−0.574	<0.001	2.690	<0.001

*Note*. MAP = mean arterial pressure, SBP = systolic blood pressure, DBP = diastolic blood pressure, *R* = multiple correlation coefficient, *β* = *β* coefficient of multiple linear regression, std. *β* = standardized *β*.

### Accuracy of MAP, SBP, and DBP estimates

**Figure 2:**
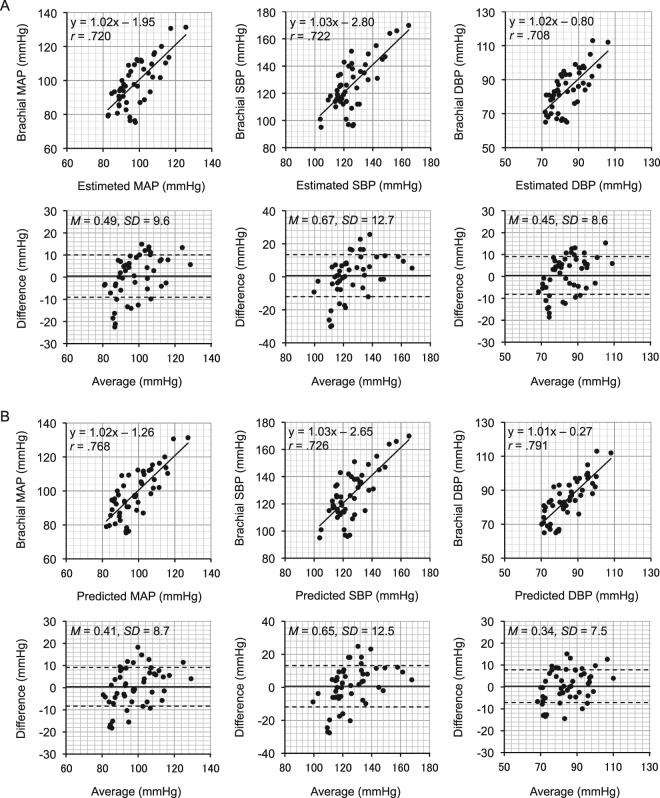
The accuracy of blood pressure estimation attained by the proposed method. (**A**) Scatterplots of mean arterial pressure (MAP: Left), systolic blood pressure (SBP: Middle), and diastolic blood pressure (DBP: Right) estimated using only the smartphone and measured using a brachial cuff sphygmomanometer (*N* = 49). (Upper) Solid line represents the regression line and its formula, together with *r* value, is shown in each scatterplot. (Lower) Corresponding Bland–Altman plots. Solid line and dashed lines represent fixed bias (*M*) and *M* ± 1 standard deviation (*SD*) range, respectively. Average = (estimate + brachial)/2, Difference = brachial – estimate. (**B**) The same analyses of (A) but using data from the dedicated photoplethysmograph (*N* = 51).

Scatterplots of paired MAP, SBP, and DBP values estimated using only the smartphone and measured using a brachial cuff sphygmomanometer, together with their Bland–Altman plots, are shown in Fig. 2(A). The same analyses using those from the dedicated photoplethysmograph are shown in Fig. 2(B). The estimated MAP, SBP, and DBP from the smartphone and the dedicated photoplethysmograph were not correlated with their residuals, respectively (all |*r*|s < 0.03). Shapiro-Wilk tests did not detect a strong violation of normality of the distribution in the residuals (all *p*s > 0.026).

## Discussion

The present findings offer a simple and moderately reliable cuffless method for estimating BP using only a smartphone, as well as a traditional finger photoplethysmograph. The estimated MAP, SBP, and DBP attained an accuracy of *R* > 0.70 (Fig. 2) when compared to a standard brachial cuff sphygmomanometer, across gender and across baseline and stress conditions, where pre-ejection period (PEP), one of the major error sources of the PWV-based method^12,14,16,17,39,40^, is expected to differ^41^, without relying on a complicated individual calibration procedure and on any other devices such as an ECG and dedicated PPG^11,16,21^. As predicted, judging from standardized *β* coefficients shown in Table 2, the estimated MAP depended both on HR and ln mNPV, the estimated SBP mainly depended on HR, and the DBP depended primarily on ln mNPV and secondly on HR. These findings imply that the present cuffless method could allow a huge number of smartphones all over the world^42^ to be stand-alone, simplified sphygmomanometers. This could also be true for smart watches once they become capable of measuring mNPV. Considering the necessity and the benefits of frequent BP measurements in daily life around the world^43^, even such a simplified sphygmomanometer may be expected, in the not-too-distant future, to play a certain role in preventing people from developing serious cardiovascular disease. Having said that, the present method needs further development before it can be used for clinical assessments.

The accuracy represented by *R* values > 0.70 for MAP, SBP, and DBP (Fig. 2) were, firstly, sometimes inferior to those (|*r*|s > 0.74) from PWV-based cuffless BP with calibration^11,16,19^ and sometimes superior to that (|*r*|s < 0.67) from a PWV-method where PEP is not constant^14^. However, the present method does not rely on calibration using a cuff sphygmomanometer or an ECG. Although incorporating an individual calibration process and/or PWV value into the multiple regression equation to reduce residual error would improve the estimation accuracy of our method to a certain degree, this would sacrifice its simplicity. Secondly, this accuracy is slightly superior to those (|*r*|s = 0.44–0.58) from PPG amplitude-based cuffless BP measurements^22,24^, and roughly equal to that (|*r*|s = 0.50–0.92) from PPG morphology-based cuffless BP measurements using multiple linear regression^23^, which only requires PPG. The method is the same as the present one in that both only require PPG, but the advantage of our method is that it only requires the use of a smartphone; there is no need for a data acquisition system or bio-amplifier. Thirdly, the accuracy of our method (|*r*|s = 0.48 and 0.59 for DBP and SBP, respectively) is marginally superior to that of a combined PWV–PPG-based method with machine learning using PPG feature extraction^21^. While this combined method does not require individual calibration, it still requires an ECG. Taken together, the present method is simple to use and relatively accurate.

All multiple correlation coefficients, *R* values, from a smartphone were slightly lower than those from a laboratory photoplethysmograph. In addition, all multiple regression coefficients, c, from the smartphone were higher than those from the dedicated photoplethysmograph. This might be due to the wavelength of the light used in the finger PPG; green for the smartphone, and near infrared for the laboratory photoplethysmograph. Near-infrared light has sufficient penetration depth to probe peripheral arterioles where the blood pressure is equal to that in the brachial artery^5,44^. In contrast, green light has a much shallower penetration depth compared to near-infrared light, and consequently probes more downstream arterioles near blood capillaries where the blood pressure decreases^44^. Thus, decrease in pressure is most likely linked to the higher c coefficients in the smartphone. Moreover, there is no guarantee that these decreases in BP are constant across participants or across MAP, SBP, and DBP. Thus, this variation plausibly results in lower *R* values.

The *R* values might be better by using higher order regression. Although “models with degree higher than 2 are rarely required in practice”^45^, inverse U-shape relationships are often observed between CO and HR e.g.^46^. So, we have tried testing quadratic models. However, due to quite high multicollinearity (in the current data set, *r* values between HR and HR-squared are over 0.99), regression analyses were not stable, such as resulted not in inverse U-shape as expected but in U-shape relationship and in non-significant *β* coefficients either in HR or HR-squared by cancelling out each other, in spite of virtually no increase in *R* values. Considering the fact that the literature sometimes failed to replicate such quadratic relationships e.g.^47^, and “likelihood surface is nearly flat near the maximum”^45^ in regression models, situations where we can take advantage of U-shape curve or higher order regression might be very limited, contrary to expectations.

We removed three data points from the iPhone data according to its auto analysis algorithm, although there was no need to remove data in our previous study where a similar stress task was used^33,35^. This difference was most likely due, firstly, to the position of the finger photoplethysmograph sensors. In the previous studies, the sensors were attached on opposite sides of the finger, whereas they were attached on the same side of the neighbouring finger in the present study. Secondly, the difference may have been a result of the smartphone size. A larger smartphone was used in the present study. The placement of the sensors and the size of the device made it harder for the participants to hold the smartphone firmly. For the removed data, the root mean square errors of the delta difference in HR and ln mNPV between the laboratory photoplethysmograph and smartphone were 12.0 and 0.56, respectively. Without removing these values from the smartphone dataset, multiple regression analysis revealed that *R* values were decreased by approximately 0.05. Although these data indicate that the auto analysis algorithm works effectively to remove aberrant values, every effort should be paid to keep the fingertip firmly on the CMOS camera of the smartphone.

Our study has several limitations. Firstly, we used a standard brachial oscillometric sphygmomanometer as a reference, so the beat-by-beat estimated MAP, SBP, and DBP from the smartphone and dedicated photoplethysmograph were not validated. Although it is known that continuous BP monitors such as those that use the vascular unloading technique, sometimes produce inaccurate values^48^, further studies using an improved continuous monitor e.g.^5,49,50^, are needed. Secondly, our participants were limited to young Japanese men and women. Thus, further studies examining whether the multiple correlation coefficients and *β* coefficients differ depending on the population are needed. Thirdly, we have used resting BL and stressful MA, but have not used other situations, including other stressful tasks, changes in body posture, and exercise. Future studies incorporating these conditions are desirable, as only a few studies have investigated more than three conditions together e.g.^11,14^.

Despite these limitations, we offer a simple and moderately reliable method for estimating MAP, SBP, and DBP using only a smartphone without using a complicated calibration procedure requiring cuff sphygmomanometer measurements and any other devices, such as an ECG and dedicated PPG. The present method could potentially convert a huge number of smartphones into simplified sphygmomanometers.

## Methods

### Participants

A total of 13 volunteers [six Japanese women and seven Japanese men; aged 20–24 years; body mass index = 20.4 ± 1.4 (mean ± standard deviation); at least 12 years of education; and living in Sapporo City], recruited via flyers placed around the university, participated in this study. According to the 2017 ACC/AHA classification^51^, six out of 13 participants were classified as normal, zero was elevated, four were hypertension stage 1, and three were hypertension stage 2. Because the present method is new and no direct effect size was available from previous studies, we reviewed studies examining PWV-based BP and found that a relatively small sample size was acceptable unless there was a special reason for a large sample size, such as using machine learning. For example, Payne *et al*.^14^ used *N* = 12, Douniama *et al*.^39^ used *N* = 14, and Patzak *et al*.^52^ used *N* = 12. Based on these studies, we adopted a relatively small sample size *N* = 13. The criteria for inclusion in the study were being over the age of 20, having no current cardiovascular disease except for high blood pressure, and not taking any prescription medication. The participants received approximately US$20 for their participation. Written informed consent was obtained from participants after we had provided them with a complete description of the study. This study was approved by the ethics committee of Hokkaido University and conducted according to the principles expressed in the Declaration of Helsinki. This is not a replicated study.

### Apparatus and measurements

#### Smartphone

Finger PPG was measured from the left index finger using a smartphone (Apple, iPhone 6s), within which the iPhysioMeter^SM^ app (version 2.0) was installed. iPhysioMeter^SM^ is a software program that was designed to run on iPhone 5 s and later smartphones running with iOS 9.2 or later (Apple) and to allow a smartphone to be a reflectance mode photoplethysmograph using the built-in LED flash light and built-in CMOS camera as a light source and photodetector, respectively. The latest version of iPhysioMeter^SM^ has a 60 Hz (frames per second; fps) sampling speed, which is twice as high as the previous version, and has been equipped with newly designed digital filters.

The beat-by-beat HR and ln mNPV were derived using an auto analysis function equipped with iPhysioMeter^SM^ *ab initio*, and show high agreement with those derived from a dedicated photoplethysmograph. Values departing significantly from the preceding 10-s period, defined as those contributing to an increase in the SD of the period above 8.0 beats per minute (bpm) for HR or 0.25 arbitrary units (a.u.) for ln mNPV, respectively, are judged as outliers^33,34^.

#### Laboratory device

Finger PPG was measured using a transmittance mode photoplethysmograph with an 810-nm, near-infrared light-emitting diode (LED; Ushio Opto Semiconductors, SMC810) as a light source and a photodiode (OSRAM, BPW34FAS) as a photosensor, placed on opposite sides of the tip of the left middle finger, as previously reported^33,35^. By means of a bio-amplifier, finger PPG was recorded using an A/D converter (National Instruments, USB-6211) at a rate of 1 kHz with a resolution of 16 bits, and stored digitally in a virtual windows-based computer (Apple, MacBook Pro, Retina 15-inch Mid 2012).

The beat-by-beat HR was derived by dividing 60,000 ms by the inter-beat interval (ms) of the alternating-current (AC) component of the finger PPG. mNPV, an index of α-adrenalin-mediated sympathetic activity^30–32^, was calculated by dividing the AC amplitude (mV) of the finger PPG by their corresponding direct-current (DC) component (mV).

#### Reference

SBP and DBP were measured using a brachial cuff sphygmomanometer (NISSEI, DS-S10) attached to the right arm. MAP was calculated using the following formula: MAP = DBP + (SBP − DBP)/3.

### Procedure

The experiment was performed in a 4 × 5 m conference room maintained at a temperature of 24−26 °C. After the sensor of the finger photoplethysmograph and the brachial cuff of the sphygmomanometer were attached, the participants sat in a chair with both of their hands on a desk and holding a smartphone in their left hand while keeping their index finger on the CMOS camera. The brachial cuff and fingers were supported at heart level. Each participant was instructed to keep as still as possible until the end of the experiment in order to minimize movement artifacts.

**Figure 3:**
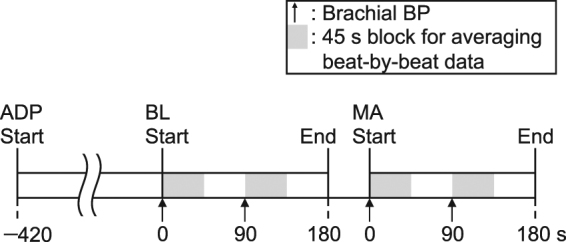
Experimental procedure. After 7-min adaptation (ADP), 3-min baseline (BL) was followed by 3-min mental arithmetic (MA) period. Arrows represent the start of each measurement using the brachial cuff sphygmomanometer. Each halftone screening period represents a 45-s block for averaging the beat-by-beat data.

The experiment began with a 7-min adaptation, which was followed by a 3-min baseline (BL), as shown in Fig. 3. Finger PPGs from the smartphone and the dedicated device were measured simultaneously during the BL period. Brachial BP measurements were carried out during the first and the third quarters of the BL (i.e., 0–45 s and 90–135 s, respectively). Next, the participants performed a 3-min mental arithmetic task (MA) where they were required to subtract 13 sequentially from 5,000 (thus, 4,987; 4,974; 4,961; …) as quickly and accurately as possible^35^. Our previous research^41^ has shown that this MA causes a decrease in PEP. Similarly to BL, both finger PPGs were measured across MA, brachial BP measurements were made during the first and the third quarters of the MA.

### Data analysis

Analyses were conducted separately but in parallel for the smartphone and the dedicated photoplethysmograph.

The beat-by-beat HR and ln mNPV were averaged for each quarter period (i.e., 45 s). The averages derived from smartphone data including over 20% outliers judged by the auto analysis routine of the iPhysioMeter^SM^ were treated as missing values. Because blood pressure was mainly measured within the first and the third quarters of BL and MA, only these averages were forwarded for further analysis.

To gain a perspective of the data, physiological values from BL and MA were averaged for each period to produce single BL and MA values, and differences between conditions were compared using a paired two-sided *t*-test.

To examine the agreement of HR and mNPV measurements derived from the smartphone and from the dedicated photoplethysmograph, geometric mean regression analysis^53^ and Bland–Altman analysis^54^ were conducted.

Multiple linear regression analyses, applying brachial ln MAP, ln SBP, and ln DBP as each dependent variable and ln HR and ln mNPV as dependent variables, were conducted.

1 $${\rm{Brachial}}\,\mathrm{ln}\,{\rm{MAP}}={{\rm{a}}}_{{\rm{MAP}}}\times \,\mathrm{ln}\,{\rm{HR}}+{{\rm{b}}}_{{\rm{MAP}}}\times \,\mathrm{ln}\,{\rm{mNPV}}+{{\rm{c}}}_{{\rm{MAP}}}$$

2 $${\rm{Brachial}}\,\mathrm{ln}\,{\rm{SBP}}={{\rm{a}}}_{{\rm{SBP}}}\times \,\mathrm{ln}\,{\rm{HR}}+{{\rm{b}}}_{{\rm{SBP}}}\times \,\mathrm{ln}\,{\rm{mNPV}}+{{\rm{c}}}_{{\rm{SBP}}}$$

3 $${\rm{Brachial}}\,\mathrm{ln}\,{\rm{DBP}}={{\rm{a}}}_{{\rm{DBP}}}\times \,\mathrm{ln}\,{\rm{HR}}+{{\rm{b}}}_{{\rm{DBP}}}\times \,\mathrm{ln}\,{\rm{mNPV}}+{{\rm{c}}}_{{\rm{DBP}}}$$

Once a, b, and c for each MAP (a_MAP_ , b_MAP_ , and c_MAP_), SBP (a_SBP_ , b_SBP_ , and c_SBP_), and DBP (a_DBP_, b_DBP_ , and c_DBP_) were derived, each estimated MAP, SBP, and DBP was calculated using the following exponential transformation (exp) equations:

4 $${\rm{Estimated}}\,{\rm{MAP}}=\exp \,({{\rm{a}}}_{{\rm{MAP}}}\times \,\mathrm{ln}\,{\rm{HR}}+{{\rm{b}}}_{{\rm{MAP}}}\times \,\mathrm{ln}\,{\rm{mNPV}}+{{\rm{c}}}_{{\rm{MAP}}})$$

5 $${\rm{Estimated}}\,{\rm{SBP}}\,=\,\exp ({{\rm{a}}}_{{\rm{SBP}}}\,\times \,\mathrm{ln}\,{\rm{HR}}\,+\,{{\rm{b}}}_{{\rm{SBP}}}\,\times \,\mathrm{ln}\,{\rm{mNPV}}\,+\,{{\rm{c}}}_{{\rm{SBP}}})$$

6 $${\rm{Estimated}}\,{\rm{DBP}}\,=\,\exp ({a}_{DBP}\,\times \,\mathrm{ln}\,{\rm{HR}}\,+\,{{\rm{b}}}_{{\rm{DBP}}}\,\times \,\mathrm{ln}\,{\rm{mNPV}}\,+\,{{\rm{c}}}_{{\rm{DBP}}})$$

To evaluate the prediction accuracy, scatterplots of MAP, SBP, and DBP between brachial and estimated values were drawn. Simple linear regression analyses, together with, Bland–Altman analysis^54^, were conducted.

All analyses were carried out using IBM SPSS Statistics 19.0 (IBM).

### Data Availability

The data that support the findings of this study are available via e-mail from the corresponding author upon reasonable request.

### Code Availability

The iPhysioMeter^SM^ App (version 2.0) is available for free at iTunes App Store (Apple).
